# Plasma Biomarkers of Alzheimer Disease in Women With and Without HIV

**DOI:** 10.1001/jamanetworkopen.2023.44194

**Published:** 2023-11-29

**Authors:** Xuantao Li, Recai Yucel, Helene Clervius, Kundun Kamalakar, Henrik Zetterberg, Kaj Blennow, Jinbing Zhang, Adaora Adimora, Lauren Collins, Margaret Fischl, Seble Kassaye, Pauline Maki, Eric Seaberg, Anjali Sharma, David Vance, Deborah R. Gustafson

**Affiliations:** 1 Department of Epidemiology and Biostatistics, Temple University, Philadelphia, Pennsylvania; 2 Department of Neurology, State University of New York Downstate Health Sciences University, Brooklyn; 3 Downstate Neurology at One Brooklyn Health, Brookdale Hospital, Brooklyn, New York; 4 School of Public Health, State University of New York Downstate Health Sciences University, Brooklyn; 5 Department of Psychiatry and Neurochemistry, Institute of Neuroscience and Physiology, the Sahlgrenska Academy at the University of Gothenburg, Mölndal, Sweden; 6 Clinical Neurochemistry Laboratory, Sahlgrenska University Hospital, Mölndal, Sweden; 7 Department of Neurodegenerative Disease, UCL Institute of Neurology, Queen Square, London, United Kingdom; 8 UK Dementia Research Institute at UCL, London, United Kingdom; 9 Hong Kong Center for Neurodegenerative Diseases, Clear Water Bay, Hong Kong, China; 10 Wisconsin Alzheimer’s Disease Research Center, University of Wisconsin School of Medicine and Public Health, University of Wisconsin, Madison; 11 Bloomberg School of Public Health, Johns Hopkins University, Baltimore, Maryland; 12 Department of Medicine, School of MedicineUniversity of North Carolina at Chapel Hill; 13 Department of Epidemiology, Gillings School of Global Public Health, University of North Carolina at Chapel Hill; 14 Division of Infectious Diseases, Emory University, Atlanta, Georgia; 15 Division of Infectious Diseases, Department of Medicine, University of Miami, Miami, Florida; 16 Department of Medicine, Division of Infectious Diseases, Georgetown University Medical Center, Washington, DC; 17 Department of Psychiatry, University of Illinois College of Medicine, Chicago; 18 Department of Psychology, University of Illinois College of Medicine, Chicago; 19 Department of Obstetrics and Gynecology, University of Illinois College of Medicine, Chicago; 20 Division of General Internal Medicine, Albert Einstein College of Medicine, New York, New York; 21 Division of Infectious Diseases, Albert Einstein College of Medicine, New York, New York; 22 Department of Acute, Chronic and Continuing Care, University of Alabama at Birmingham

## Abstract

**Importance:**

Blood-based biomarkers associated with increased risk of Alzheimer disease (AD) are understudied in people living with and without HIV, particularly women.

**Objective:**

To determine whether baseline or 1-year changes in plasma amyloid-β40 (Aβ40), Aβ42, ratio of Aβ42 to Aβ40, total tau (t-tau), phosphorylated tau 231 (p-tau_231_), glial fibrillary acidic protein (GFAP), and/or neurofilament light chain (NFL) are associated with neuropsychological performance (NP) among women living with HIV (WLWH) and women living without HIV (WLWOH).

**Design, Setting, and Participants:**

This longitudinal, prospective, cohort study with 1-year repeated clinical measures (NP only measured once) and biospecimen collection occurred between 2017 and 2019. Participants were women aged 40 years or older from 10 clinical research sites in cities across the US that were part of the Women’s Interagency HIV Study. Data analysis was conducted from April to December 2022.

**Exposure:**

Laboratory-confirmed HIV status and AD biomarkers.

**Main Outcomes and Measures:**

Sociodemographically adjusted NP T-scores (attention and working memory, executive function, processing speed, memory, learning, verbal fluency, motor function, and global performance) were the primary outcomes. Baseline and 1-year fasting plasma Aβ40, Aβ42, t-tau, p-tau_231_, GFAP, and NFL levels were measured and analyzed using multivariable linear regression.

**Results:**

The study consisted of 307 participants (294 aged ≥50 years [96%]; 164 African American or Black women [53%]; 214 women with a high school education or higher [70%]; 238 women who were current or former smokers [78%]; and 236 women [77%] who were overweight or obese [body mass index >25]) including 209 WLWH and 98 WLWOH. Compared with WLWOH at baseline, WLWH performed worse on learning (mean [SD] T*-*score 47.8 [11.3] vs 51.4 [10.5]), memory (mean [SD] T-score 48.3 [11.6] vs 52.4 [10.2]), verbal fluency (mean [SD] T-score 48.3 [9.8] vs 50.7 [8.5]), and global (mean [SD] T-score 49.2 [6.8] vs 51.1 [5.9]) NP assessments. Baseline median Aβ40, GFAP, and NFL levels were higher among WLWH vs WLWOH. There were no differences in 1-year biomarker change by HIV serostatus. Lower learning, memory, and motor NP were associated with 1-year Aβ40 increase; lower learning and motor with Aβ42 increase; lower motor with p-tau_231_ increase; and lower processing speed, verbal fluency and motor with NFL increase in the entire sample. Among WLWH, a 1-year increase in Aβ40 from baseline to follow-up was associated with worse learning, memory, and global NP; a 1-year increase in t-tau with worse executive function; and a 1-year increase in NFL with worse processing speed. Among WLWOH, a 1-year increase in Aβ40 and Aβ42 were associated with poorer memory performance and NFL was associated with poorer motor performance.

**Conclusions and Relevance:**

These findings suggest that increases in certain plasma AD biomarkers are associated with NP in WLWH and WLWOH and may be associated with later onset of AD, and measuring these biomarkers could be a pivotal advancement in monitoring aging brain health and development of AD among women with and without HIV.

## Key Points

**Question:**

Are blood-based biomarkers for Alzheimer disease (AD) associated with neuropsychological performance in women with and without HIV who are also sociodemographically underrepresented in research?

**Findings:**

This cohort study of 294 women with HIV and 98 women without HIV found that cross-sectional and 1-year changes in biomarkers of amyloid-β (Aβ), tau, and neurodegeneration (Aβ40, Aβ42, total-tau, phosphorylated tau 231, and neurofilament light chain) were associated with domain-specific neuropsychological performance.

**Meaning:**

These findings suggest that measuring blood-based biomarkers associated with neuropsychological performance could be a pivotal advancement in monitoring aging brain health and development of AD among women with and without HIV.

## Teaser

This cohort study analyzes whether biomarkers for Alzheimer disease are associated with neuropsychological performance among women with and without HIV.

## Introduction

Chronic HIV is a 21st century phenomenon due to effective antiretroviral therapies (ARTs) and increasing life expectancies among people living with HIV (PLWH) and fewer HIV-related deaths.^1^ Still, HIV constitutes a public health concern with more than 38 million cases worldwide.^2^ As a multisystem disorder, HIV directly and indirectly affects the peripheral and central nervous systems. PLWH may experience wide-ranging neurological disorders (eg, peripheral neuropathy and cognitive impairment) and neuropsychiatric conditions including depression.^3^ Although the incidence of HIV-associated neurocognitive disorder has decreased in PLWH due to effective and quickly prescribed ARTs,^4^ those surviving to older ages are at risk for Alzheimer disease (AD).^5^

AD is a global health concern with more than 50 million people affected worldwide.^5^ In the US, more than 6 million people aged 65 years or older live with dementia. It is projected that this number will double by 2060 and comprise more than 3% of the US population.^5^ AD is the seventh leading cause of death in the US^5^ and globally.^6^ Certain populations may be more susceptible to AD, notably, those with multiple comorbidities and African American or Black people.^5^ Besides reducing quality of life, AD has a major economic impact due to caregiving and prohibitive costs of symptom treatment, which has reached $60 billion.^5^ Although there is no cure for AD, early diagnosis is critical for appropriate health and care management and caregiver and family planning.

Approximately 50% of PLWH, whether treated with ARTs or not, experience a cognitive disorder.^7^ Women living with HIV (WLWH) and women living without HIV (WLWOH), may be at risk for more adverse brain health outcomes with aging due to higher levels of vascular risk factors, such as hypertension, obesity, and cigarette smoking, as well as substance abuse, lower education, lower income, and inadequate health care access.^5^

Blood-based AD biomarkers reflecting amyloid, tau, and neurodegeneration (ATN) may be keys to early diagnosis and underlie the National Institute on Aging and Alzheimer’s Association 2018 research framework^8^; the framework mandates research based on ATN, with or without clinical manifestations, to better understand the mechanisms underlying AD, the temporal sequence of AD neuropathologies, and the temporality of factors associated with increased risk of AD. Thus, novel blood-based AD biomarkers include those reflecting amyloid deposition (eg, amyloid-β [Aβ] 40, Aβ42, and the Aβ42:Aβ40 ratio), tau pathophysiology (eg, total tau [t-tau] and phosphorylated tau231 [p-tau_231_]), and neurodegeneration (eg, neurofilament light chain [NFL] and glial fibrillary acidic protein [GFAP]).^9^ These biomarkers will complement and/or eventually confirm a clinical AD diagnosis based on the ATN criteria^10^ and contribute to better understanding of ATX(N),^11^ where the X represents other pathological factors, such as neuroinflammation, immune function, vascular events, and HIV. Thus, identification of blood-based biomarkers of AD against a background of chronic infectious disease and vascular risk is paramount to understanding HIV and ARTs in relation to cognitive impairments and, eventually, late-onset AD.^12^

Given the dearth of observational, longitudinal data on blood-based AD biomarkers in WLWH, we explored the hypothesis: is 1-year change in plasma AD biomarkers associated with neuropsychological performance (NP) among WLWH and WLWOH aged 40 years or older at baseline? The Women’s Interagency HIV Study (WIHS) extant data set^13^ and stored biospecimens were used.

## Methods

### Study Population and Setting

This cohort study followed the Strengthening the Reporting of Observational Studies in Epidemiology (STROBE) reporting guideline; the institutional review boards of each clinical research site approved the WIHS research protocol, and all participants provided written informed consent. The WIHS was the largest prospective study of HIV in women (defined as sex at birth and gender) in the US.^13^ WIHS began in 1994 and initially enrolled 2054 WLWH and 1712 WLWOH who were sociodemographically similar across 6 sites in San Francisco, California; Los Angeles, California; Chicago, Illinois; Washington, DC; and Brooklyn and the Bronx in New York, New York. Two additional enrollment waves occurred in 2001 to 2002 and 2011 to 2014. In 2012, 4 additional sites were added in Atlanta, Georgia; Birmingham and Jacksonville, Alabama; Chapel Hill, North Carolina; and Miami, Florida; and 1 site was dropped (Los Angeles, California). Semiannual WIHS core visits included sociodemographic, behavioral, and clinical measures. A standardized comprehensive NP battery was administered every 2 years starting in 2009. A subset of WIHS participants who completed 1 NP assessment between 2017 and 2019 had batch testing of fasting plasma Aβ40, Aβ42, t-tau, p-tau_231_, GFAP, and NFL levels in 2018 (designated as baseline) and 1 year later in 2019.

### Sociodemographic and Health History Measures

Sociodemographic factors were self-reported and included date of birth, race (survey options included African American or Black, White, and other race [defined as American Indian or Native Alaskan, Asian, Native Hawaiian or Pacific Islander, and multiracial]), and ethnicity (Hispanic or non-Hispanic). Race and ethnicity were self-reported and queried according to US Census definitions; race and ethnicity data were included in this study because NP T-scores were adjusted for race and ethnicity. Participants were also asked about the highest educational level attained, annual income, use of tobacco, marijuana, alcohol, and other drugs (crack, cocaine, or heroin). They were also asked about their history of hypertension, use of hypertensive medications, history of diabetes, and use of diabetes medications.

### Clinical Measures

Body weight and body height measures were measured with participants wearing undergarments.^14^ Body mass index (BMI) was calculated as weight in kilograms divided by height in meters squared. Categories of BMI included underweight (<18.5), healthy weight (18.5–24.9), overweight (25.0–29.9), and obese (≥30).^15^

Systolic blood pressure and diastolic blood pressure were recorded using a standardized protocol with the mean of multiple resting measurements used for analysis. Hypertension was defined as the use of prescribed antihypertensive medications. Diabetes was determined as fasting glucose greater than or equal to 126 mg/dL (to convert glucose to mmol/L, multiply by 0.0555) hemoglobin A_1C_ greater than or equal to 6.5% (to convert hemoglobin A_1C_ to the proportion of total hemoglobin, multiply by 0.01), or self-reported use of diabetes medications.^16^ Estimated glomerular filtration rate (eGFR) was calculated using the chronic kidney disease epidemiology equation.^17^

### Neurological and Mental Health Measures

Neurological measures included self-reported memory loss, numbness or tingling of the hands and feet, confusion and getting lost, and depression using the Center for Epidemiological Studies-Depression (CES-D) scale.^18^ The presence of clinically relevant depressive symptoms was defined as a CES-D score of 16 or greater of 60.

### HIV-Related Measures

Laboratory-confirmed HIV status, HIV viral load, and CD4 T cell count were considered in analyses of WLWH. Self-reported ART use, and AIDS diagnosis were considered in analyses of WLWH.

### NP Outcomes

The NP battery included the Letter-Number Sequencing test,^19,20^ Trail Making Test Part B,^21^ Stroop Test (color word and word reading),^22^ Hopkins Verbal Learning Test-Revised (HVLT-R),^23^ Symbol Digit Modalities Test,^24,25^ Controlled Oral Word Association Test,^26^ Category Fluency Test (Animals),^27^ and Grooved Pegboard Test.^28^ Performance on these assessments was used to assess 7 NP domains: (1) attention and working memory (outcomes: total correct on the Letter-Number Sequencing control and experimental conditions), (2) executive function (outcomes: time to completion on the Trail Making Test Part B and Stroop color word test [interference] trial), (3) processing speed (outcomes: total correct on the Symbol Digit Modalities Test and time to completion on the Stroop word-reading trial), (4) memory (outcome: HVLT-R delayed recall score), (5) learning (outcome: total learning across HVLT-R trials), (6) verbal fluency (outcomes: total correct on Controlled Oral Word Association Test and Category Fluency Animals test); and (7) fine motor skills (outcomes: total time to completion for each hand on the Grooved Pegboard test).^29,30^

All timed outcomes were natural log-transformed and reverse scored, so that higher scores represented better performance. Cohort-derived, sociodemographically adjusted T-scores were derived for each outcome according to data for WLWOH.^30^ Sociodemographic factors included age, education, Wide Range Achievement Test reading subtest (WRAT-3) score, race (Black or African American compared with White individuals and individuals of other races), and ethnicity (Hispanic and non-Hispanic). T-scores were calculated to create domain-specific and global NP scores.^29,30^ Global NP score was the calculated mean of the 7 NP domains.

### AD Biomarker Measures

Baseline and 1-year fasted plasma Aβ40, Aβ42, t-tau, p-tau_231_, GFAP, and NFL concentrations were measured in batch from stored EDTA plasma samples using single molecule array (Simoa) technology on an HD-X Analyzer (Quanterix). Plasma Aβ40, Aβ42, GFAP, and NFL concentrations were measured using the Neuro 4-Plex E kit according to instructions from the manufacturer (Quanterix). Plasma t-tau concentration was measured using the Tau Advantage assay kit. Plasma p-tau_231 _concentration was measured using an in-house Simoa assay as previously described.^31^ Standards and controls were tested in duplicate. Paired longitudinal samples were run side by side on the same plates. The measurements were performed in 1 round of experiments using 1 batch of kits. Intraassay coefficients of variation were below 5% for all biomarkers, except for t-tau where intraassay coefficients of variation were 3.9% to 6.8%.

### Statistical Analysis

Participants were characterized overall and by HIV serostatus. Covariates of interest included sociodemographic, behavioral, clinical and neurological factors. Among WLWH, HIV viral load, CD4 T cell count, and history of AIDS were characterized. We calculated *t* tests for continuous variables and χ^2^ tests for categorical variables to assess differences by HIV status at baseline. Primary independent variables were baseline blood-based AD biomarker levels and 1-year change in biomarker levels. One-year change was calculated as baseline biomarker level subtracted from follow-up biomarker level. The primary outcomes were domains of NP and global NP. Multivariable linear regression analyses were performed to examine cross-sectional baseline and 1-year change in level of each plasma AD biomarker in association with the outcome and NP T-scores among all women (adjusted for HIV serostatus) and stratified by HIV serostatus. In analyses of WLWH only, we additionally adjusted for baseline HIV viral load, CD4 T cell count category, and history of AIDS.

Covariates were selected on the basis of previous WIHS analyses and included income, BMI, CES-D score, history of diabetes, use of hypertensive medication, alcohol, tobacco, marijuana, and other drug use (crack, cocaine, or heroin).^14,29,32^ We also adjusted for eGFR because kidney function may influence some of these biomarker levels.^33^ Race, ethnicity, WRAT score, education, and age were not included as covariates because they were already included in the demographically adjusted NP T-scores. Data analyses were conducted using R software version 4.3.2 (R Project for Statistical Computing). Results were considered significant at 2-sided *P* < .05. Data analysis was conducted from April to December, 2022.

## Results

The study pool consisted of 307 women (294 aged ≥50 years [96%]; 135 aged ≥60 years [44%]; 16 aged ≥70 years [5%]; 164 African American or Black women [53%]), including 209 WLWH and 98 WLWOH. Most women had a high school education or higher (214 participants [70%]), were current or former smokers (238 participants [78%]), or had overweight or obesity (BMI ≥25.0, 236 participants [77%]). Compared with WLWOH, WLWH performed more poorly on learning (mean [SD] T*-*score 47.8 [11.3] vs 51.4 [10.5]), memory (mean [SD] T*-*score 48.3 [11.6] vs 52.4 [10.2]), verbal fluency (mean [SD] T*-*score 48.3 [9.8] vs 50.7 [8.5]), and global (mean [SD] T*-*score 49.2 [6.8] vs 51.1 [5.9]) NP assessments. Baseline survey and clinical characteristics of participants are presented in Table 1. No participants had a diagnosis of AD or other type of dementia.

**Table 1.** Baseline Demographic, Alzheimer Disease Biomarker, and Neuropsychological Performance Characteristics for All Women and by HIV Serostatus

Characteristic	Participants, No. (%)^a^			*P* value
	All (N = 307)	WLWH (n = 209)	WLWOH (n = 98)	
Age range, y				
40-49	13 (4)	0	13 (13)	<.001
50-59	159 (52)	108 (52)	51 (52)	
≥60	135 (44)	101 (48)	34 (35)	
Race				
African American or Black	164 (53)	111 (53)	53 (54)	.04
White	35 (11)	30 (14)	5 (5)	
Other^b^	108 (35)	68 (33)	40 (41)	
Ethnicity				
Hispanic	65 (21)	43 (21)	22 (22)	.82
Non-Hispanic	242 (79)	166 (79)	76 (78)	
Education				
Less than high school	93 (30)	63 (30)	30 (31)	>.99
High school or higher	214 (70)	146 (70)	68 (69)	
Annual income, $				
≤12 000	154 (50)	103 (49)	51 (52)	.68
>12 000	152 (50)	106 (51)	46 (47)	
Tobacco use				
Never smoker	69 (22)	53 (25)	16 (16)	.11
Current or former smoker	238 (78)	156 (75)	82 (84)	
Marijuana use				
Never smoker	253 (82)	178 (85)	75 (77)	.09
Current or former smoker	54 (18)	31 (15)	23 (23)	
Drinking history				
Abstainer or none	164 (53)	118 (56)	46 (47)	.19
Low to moderate	133 (43)	86 (41)	47 (48)	
High	10 (3)	5 (2)	5 (5)	
Other drug use (crack, cocaine, or heroin)				
Yes	18 (6)	11 (5)	7 (7)	.69
No	289 (94)	198 (95)	91 (93)	
Hypertension				
Yes	166 (54)	117 (56)	49 (50)	.83
No	121 (39)	83 (40)	38 (39)	
Type 2 diabetes				
Yes	101 (33)	67 (32)	34 (35)	.65
No	206 (67)	142 (68)	64 (65)	
Body mass index^c^				
Healthy (18.5-24.9)	69 (22)	51 (24)	18 (18)	.001
Overweight (25.0-29.9)	94 (31)	75 (36)	19 (19)	
Obese (≥30)	142 (46)	83 (40)	59 (60)	
Estimated glomerular filtration rate, mL/min/1.73 m^2^				
<30	13 (4)	8 (4)	5 (5)	.02
30-44.9	12 (4)	7 (3)	5 (5)	
45-59.9	33 (11)	30 (14)	3 (3)	
≥60	249 (81)	164 (78)	85 (87)	
Neurological measures				
Memory loss				
Yes	21 (7)	13 (6)	8 (9)	.70
No	286 (93)	196 (94)	90 (92)	
Numbness or tingling hands and feet				
Yes	69 (22)	42 (20)	27 (28)	.19
No	238 (78)	167 (80)	71 (72)	
Confusion or getting lost				
Yes	12 (4)	9 (4)	3 (3)	.76
No	295 (96)	200 (96)	95 (97)	
Center for Epidemiological Studies-Depression scale score				
<16	225 (73)	157 (75)	68 (69)	.43
≥16	81 (26)	52 (25)	29 (30)	
HIV variables				
CD4 T cell count, cells/mL			-	
<200	NA	10 (5)	NA	NA
200-499	NA	148 (71)	NA	
≥500	NA	51 (24)	NA	
HIV RNA viral load, copies/mL				
Viral load undetectable (<80)	NA	181 (87)	NA	NA
Viral load detectable (≥80), median (IQR)	NA	1130 (188-6270)	NA	
Taking antiretroviral therapy				
Yes	NA	150 (72)	NA	NA
No	NA	58 (28)	NA	
History of AIDS				
Yes	NA	108 (52)	NA	NA
No	NA	101 (48)	NA	
Neuropsychological performance assessments, T-score, mean (SD)^d^				
Executive function	49.6 (9.4)	49.1 (9.8)	50.7 (8.2)	.15
Processing speed	49.2 (10.6)	48.8 (10.7)	50.1 (10.4)	.30
Attention	48.5 (9.3)	48.7 (9.5)	48.2 (9.0)	.68
Learning	48.9 (11.2)	47.8 (11.3)	51.4 (10.5)	.009
Memory	49.6 (11.4)	48.3 (11.6)	52.4 (10.2)	.002
Verbal fluency	49.1 (9.4)	48.3 (9.8)	50.7 (8.5)	.03
Motor	49.6 (11.6)	49.5 (11.2)	49.9 (12.6)	.80
Global	49.8 (6.6)	49.2 (6.8)	51.1 (5.9)	.02

Abbreviations: NA, not available; WLWH, women living with HIV; WLWOH, women living without HIV.

^a^ Variables reported as No. (%) were analyzed with χ^2^ tests.

^b^ Other race was defined as American Indian or Native Alaskan, Asian, Native Hawaiian or Pacific Islander, and multiracial.

^c^ Body mass index was calculated as weight in kilograms divided by height in meters squared.

^d^ T-scores are demographically adjusted for age, education, Wide Range Achievement Test reading subtest score, race (African American vs White or other race), and ethnicity (Hispanic vs non-Hispanic).

Baseline and 1-year change in biomarker characteristics are presented in Table 2. At baseline, median plasma levels of Aβ40, GFAP, and NFL were higher among WLWH compared with WLWOH. There were no differences in plasma Aβ42, ratio of Aβ42 to Aβ40, t-tau, or p-tau_231_. There were no differences in 1-year biomarker level change by HIV serostatus. Scatterplots of each biomarker at baseline and follow-up are provided as eFigures 1-6 in Supplement 1.

**Table 2.** Baseline and 1-Year Change in AD Biomarker Characteristics for All Women and by HIV Serostatus

Biomarkers	Biomarker level, median (IQR), pg/mL^a^			*P* value
	All (N = 307)	WLWH (n = 209)	WLWOH (n = 98)	
Baseline				
Aβ40	97.30 (82.70 to 111.00)	99.30 (85.80 to 113.00)	92.05 (79.33 to 104.75)	.004
Aβ42	5.96 (4.41 to 7.40)	6.06 (4.52 to 7.50)	5.70 (4.26 to 7.15)	.17
Aβ42/Aβ40 ratio	0.06 (0.05 to 0.07)	0.06 (0.05 to 0.07)	0.06 (0.05 to 0.07)	.84
GFAP	80.20 (56.25 to 118.50)	88.70 (62.10 to 127.00)	65.85 (50.15 to 97.78)	<.001
NFL	13.30 (9.59 to 20.45)	14.10 (10.00 to 22.10)	11.10 (8.61 to 15.90)	.002
T-tau	2.78 (1.93 to 3.80)	2.74 (1.89 to 3.61)	2.97 (2.03 to 3.98)	.08
P-tau_231_	5.33 (3.73 to 7.37)	5.15 (3.73 to 7.29)	5.44 (3.90 to 7.64)	.46
Change at 1 y				
Aβ40	−5.10 (−24.5 to 7.2)	−5.00 (−22.10 to 9.00)	−5.70 (−26.43 to 5.78)	.48
Aβ40	−0.40 (−1.79 to 0.64)	−0.38 (−1.80 to 0.67)	−0.48 (−1.75 to 0.49)	.34
Aβ42/Aβ40 ratio	0.00 (−0.01 to −0.00)	0.01 (−0.01 to 0.00)	−0.00 (−0.01 to 0.00)	.83
GFAP	1.00 (−13.10 to 14.80)	1.40 (−12.00 to 15.90)	−0.70 (−13.88 to 13.93)	.65
NFL	0.48 (−1.95 to 3.01)	0.52 (−2.40 to 3.10)	0.37 (−1.66 to 2.64)	.65
T-tau	−0.05 (−0.77 to 0.64)	−0.02 (−0.70 to 0.67)	−0.07 (−0.86 to 0.59)	.65
P-tau_231_	0.29 (−0.89 to 1.53)	0.38 (−0.74 to 1.55)	0.24 (−1.01 to 1.52)	.22

Abbreviations: Aβ, amyloid β; AD, Alzheimer disease; GFAP; glial fibrillary protein; NFL, neurofilament light chain; P-tau, phosphorylated tau; T-tau, total tau; WLWH, women living with HIV; WLWOH, women living without HIV.

^a^ Variables reported as median (IQR) were analyzed with the median test.

Cross-sectional analyses associating NP by baseline biomarker level showed that higher t-tau levels were associated with better learning performance among WLWH (β, 1.15; 95% CI, 0.11-2.18; *P* = .03). Among WLWOH, higher Aβ42 to Aβ40 ratio was associated with better attention (β, 137.04; 95% CI, 0.78-273.29; *P* = .049) (Table 3).

**Table 3.** Linear Regression Models Associating Cross-Sectional Baseline Plasma AD Biomarker Measures With Neuropsychological Performance by Domain in All Women and Stratified by HIV Serostatus^a^

Performance domain and AD biomarker	All participants			WLWH^b^			WLWOH		
	No.	β (95%CI)	*P* value	No.	β (95%CI)	*P* value	No.	β (95%CI)	*P* value
Executive function									
Aβ40	277	0.00 (−0.04 to 0.04)	.89	188	0.01 (−0.04 to 0.06)	.75	83	−0.03 (−0.10 to 0.04)	.37
Aβ42	275	0.06 (−0.42 to 0.54)	.81	188	0.16 (−0.45 to 0.77)	.60	81	−0.32 (−1.19 to 0.55)	.46
Aβ42/Aβ40 ratio	275	−6.21 (−82.33 to 69.91)	.87	188	18.37 (−84.26 to 120.99)	.72	81	−9.70 (−139.16 to 119.76)	.88
GFAP	277	−0.01 (−0.03 to 0.02)	.70	188	−0.01 (−0.05 to 0.02)	.41	83	−0.01 (−0.06 to 0.03)	.60
NFL	277	0.02 (−0.02 to 0.06)	.25	188	0.01 (−0.04 to 0.06)	.63	83	0.00 (−0.19 to 0.19)	.99
T-tau	277	0.02 (−0.45 to 0.48)	.95	188	0.12 (0.82 to 1.06)	.80	83	−0.23 (−0.77 to 0.31)	.40
P-tau_231_	276	0.06 (−0.14 to 0.27)	.55	187	0.11 (−0.20 to 0.43)	.47	83	−0.14 (−0.45 to 0.17)	.05
Processing speed									
Aβ40	280	0.02 (−0.03 to 0.06)	.46	189	0.04 (−0.01 to 0.09)	.11	84	−0.05 (−0.13 to 0.03)	.22
Aβ42	278	0.07 (−0.47 to 0.61)	.79	189	0.27 (−0.39 to 0.93)	.43	82	−0.48 (−1.59 to 0.64)	.40
Aβ42/Aβ40 ratio	278	−21.73 (−107.35 to 63.88)	.62	189	−13.75 (−124.65 to 97.16)	.81	82	−25.54 (−195.28 to 144.19)	.77
GFAP	280	−0.01 (−0.04 to 0.02)	.70	189	0.23 (−3.57 to 0.04)	.91	84	−0.04 (−0.10 to 0.02)	.18
NFL	280	0.03 (−0.02 to 0.07)	.25	189	0.03 (−0.03 to 0.08)	.33	84	0.04 (−0.20 to 0.28)	.74
T-tau	280	−0.11 (−0.63 to 0.41)	.67	189	−0.38 (−1.39 to 0.62)	.45	84	−0.10 (−0.81 to 0.60)	.78
P-tau_231_	279	−0.04 (−0.26 to 0.19)	.75	188	−0.02 (−0.35 to 0.31)	.90	84	−0.16 (−0.57 to 0.24)	.42
Attention									
Aβ40	263	0.01 (−0.03 to 0.04)	.64	175	0.01 (−0.03 to 0.06)	.61	81	−0.01 (−0.08 to 0.05)	.67
Aβ42	261	0.21 (−0.27 to 0.68)	.39	175	0.18 (−0.41 to 0.77)	.55	79	0.06 (−0.86 to 0.97)	.90
Aβ42/Aβ40 ratio	261	41.90 (−32.62 to 116.41)	.27	175	−2.82 (−1.01 to 9.50)	.96	79	137.04 (0.78 to 273.29)	.049
GFAP	263	0.02 (−0.01 to 0.004)	.21	175	0.02 (−0.01 to 0.06)	.16	81	−0.01 (−0.06 to 0.04)	.56
NFL	263	0.03 (0.00 to 0.07)	.07	175	0.03 (−0.02 to 0.07)	.22	81	0.07 (−0.13 to 0.27)	.47
T-tau	263	0.21 (−0.23 to 0.66)	.35	175	−0.09 (−0.97 to 0.80)	.86	81	0.44 (−0.13 to 1.01)	.13
P-tau_231_	262	0.06 (−0.13 to 0.26)	.52	174	0.08 (−0.21 to 0.37)	.59	81	−0.01 (−0.35 to 0.32)	.94
Learning									
Aβ40	280	−0.01 (−0.05 to 0.04)	.76	190	−0.01 (−0.06 to 0.04)	.82	83	0.01 (−0.08 to 0.09)	.88
Aβ42	278	−0.11 (−0.67 to 0.45)	.70	190	−0.11 (−0.80 to 0.58)	.75	81	−0.16 (−1.36 to 1.05)	.80
Aβ42/Aβ40 ratio	278	37.09 (−50.59 to 124.77)	.41	190	33.50 (−81.55 to 148.55)	.57	81	7.70 (−157.50 to 172.91)	.93
GFAP	280	0.01 (−0.02 to 0.04)	.68	190	0.02 (−0.02 to 0.05)	.43	83	−0.01 (−0.07 to 0.05)	.75
NFL	280	0.01 (−0.03 to 0.05)	.66	190	0.01 (−0.05 to 0.06)	.85	83	0.23 (−0.01 to 0.47)	.06
T-tau	280	0.31 (−0.22 to 0.84)	.25	190	1.15 (0.11 to 2.18)	.03	83	0.17 (−0.51 to 0.86)	.61
P-tau_231_	279	−0.04 (−0.27 to 0.19)	.72	189	0.01 (−0.34 to 0.35)	.96	83	−0.02 (−0.42 to 0.37)	.91
Memory									
Aβ40	280	−0.02 (−0.06 to 0.02)	.35	190	−0.03 (−0.08 to 0.06)	.30	83	0.01 (−0.07 to 0.08)	.87
Aβ42	278	−0.42 (−0.97 to 0.13)	.14	190	−0.59 (−1.27 to 0.10)	.09	81	−0.33 (−1.38 to 0.73)	.54
Aβ42/Aβ40 ratio	278	−9.14 (−97.49 to 79.22)	.84	190	−37.33 (−153.08 to 78.42)	.53	81	−47.16 (−207.81 to 113.49)	.56
GFAP	280	0.00 (−0.03 to 0.03)	.89	190	0.01 (−0.03 to 0.04)	.68	83	−0.01 (−0.07 to 0.04)	.66
NFL	280	−0.01 (0.05 to 0.04)	.80	190	−0.02 (−0.07 to 0.04)	.51	83	0.16 (−0.07 to 0.38)	.16
T-tau	280	0.24 (−0.30 to 0.77)	.39	190	0.88 (−0.17 to 1.92)	.10	83	0.10 (−0.57 to 0.78)	.76
P-tau_231_	279	−0.08 (−0.31 to 0.15)	.50	189	−0.10 (−0.45 to 0.25)	.57	83	0.01 (−0.37 to 0.40)	.95
Verbal fluency									
Aβ40	281	0.02 (−0.02 to 0.06)	.32	190	0.02 (−0.03 to 0.07)	.49	84	0.01 (−0.05 to 0.07)	.66
Aβ42	279	0.10 (−0.39 to 0.59)	.68	190	−0.00 (−0.63 to 0.63)	.10	82	0.15 (−0.69 to 0.99)	.73
Aβ42/Aβ40 ratio	279	−25.55 (−103.50 to 52.41)	.52	190	−47.43 (−152.28 to 57.43)	.37	82	−3.30 (−131.42 to 124.81)	.96
GFAP	281	−0.01 (−0.03 to 0.02)	.58	190	−0.02 (−0.06 to 0.02)	.27	84	0.01 (−0.03 to 0.06)	.54
NFL	281	0.04 (0.00 to 0.08)	.07	190	0.04 (−0.01 to 0.09)	.16	84	0.08 (−0.10 to 0.26)	.38
T-tau	281	0.09 (−0.38 to 0.57)	.71	190	−0.18 (−1.14 to 0.77)	.71	84	0.15 (−0.39 to 0.69)	.59
P-tau_231_	280	0.00 (−0.21 to 0.21)	.99	189	−0.00 (−0.32 to 0.32)	>.99	84	−0.08 (−0.39 to 0.23)	.59
Motor									
Aβ40	279	0.02 (−0.02 to 0.07)	.31	188	0.02 (−0.04 to 0.08)	.50	84	0.01 (−0.08 to 0.11)	.76
Aβ42	277	0.18 (−0.41 to 0.77)	.54	188	0.07 (−0.63 to 0.78)	.84	82	0.56 (−0.73 to 1.85)	.39
Aβ42/Aβ40 ratio	277	−14.18 (−107.98 to 79.63)	.77	188	−16.60 (−134.70 to 101.50)	.78	82	66.53 (−129.68 to 262.74)	.50
GFAP	279	−0.03 (−0.06 to 0.00)	.06	188	−0.03 (−0.07 to 0.08)	.12	84	−0.05 (−0.11 to 0.02)	.20
NFL	279	0.04 (−0.00 to 0.09)	.07	188	0.03 (−0.03 to 0.09)	.29	84	0.14 (−0.13 to 0.42)	.30
T-tau	279	0.35 (−0.22 to 0.92)	.22	188	0.51 (−0.56 to 1.58)	.35	84	0.09 (−0.74 to 0.92)	.83
P-tau231	278	0.15 (−0.10 to 0.40)	.25	187	0.26 (−0.10 to 0.61)	.15	84	−0.19 (−0.66 to 0.28)	.42
Global									
Aβ40	257	0.00 (−0.03 to 0.03)	.90	173	0.01 (−0.02 to 0.04)	.53	78	−0.03 (−0.08 to 0.02)	.19
Aβ42	255	0.05 (−0.30 to 0.40)	.79	173	0.10 (−3.38 to 0.54)	.64	76	−0.33 (−1.05 to 0.40)	.37
Aβ42/Aβ40 ratio	255	14.07 (−39.52 to 67.66)	.61	173	5.40 (−66.27 to 77.07)	.88	76	57.43 (−38.15 to 153.01)	.23
GFAP	257	0.00 (−0.02 to 0.02)	.96	173	0.01 (−0.02 to 0.03)	.68	78	−0.02 (−0.06 to 0.01)	.13
NFL	257	0.02 (−0.01 to 0.04)	.20	173	0.02 (−0.02 to 0.05)	.36	78	0.01 (−0.14 to 0.16)	.89
T-tau	257	0.11 (−0.21 to 0.43)	.49	173	0.30 (−0.34 to 0.95)	.36	78	0.01 (−0.38 to 0.39)	.97
P-tau_231_	256	−0.02 (−0.16 to 0.12)	.74	172	0.01 (−0.21 to 0.22)	.94	78	−0.13 (−0.35 to 0.09)	.25

Abbreviations: Aβ, amyloid β; AD, Alzheimer disease; GFAP, glial fibrillary protein; NFL, neurofilament light chain; P-tau, phosphorylated tau; T-tau, total tau; WLWH, women living with HIV; WLWOH, women living without HIV.

^a^ All models are adjusted for HIV status, income, body mass index, Center for Epidemiological Studies-Depression scale score, estimated glomerular filtration rate, history of hypertension, diabetes, tobacco use, marijuana use, alcohol use, and other drug use (crack, cocaine, or heroin). β represents an adjusted, unstandardized coefficient. Neuropsychological performance assessments were reverse scored, and a higher β indicates better neuropsychological performance.

^b^ WLWH models were additionally adjusted for baseline CD4 T cell count, HIV viral load, and history of AIDS.

One-year biomarker change calculated as baseline subtracted from follow-up (Table 4), indicated the following among all women, adjusted for HIV status and other covariates: a greater average increase in Aβ40 was associated with worse learning, memory, and motor performance; greater increase in Aβ42 with worse learning and motor performance; greater increase in p-tau_231 _with worse motor performance; and greater increase in NFL level with worse processing speed, verbal fluency, and motor performance. When stratified by HIV status, associations of biomarkers with NP predominated in WLWH. Among WLWH, a greater average increase in Aβ40 level was associated with worse learning, memory, and global NP; a greater increase in t-tau with worse executive function; and a greater decrease in NFL level with worse processing speed. Among WLWOH, greater increases in Aβ40 and Aβ42 levels were associated with poorer memory performance, and a greater increase in NFL level with poorer motor performance.

**Table 4.** Linear Regression Models Associating 1-Year Change in AD Biomarker Measures With Neuropsychological Performance by Domain in All Women and by HIV Serostatus^a^

Performance domain and AD biomarker	All participants			WLWH^b^			WLWOH		
	No.	β (95%CI)	*P* value	No.	β (95%CI)	*P* value	No.	β (95%CI)	*P* value
Executive function									
Aβ40	275	−0.00 (−0.04 to 0.03)	.90	188	−0.01 (−0.06 to 0.03)	.54	81	0.02 (−0.05 to 0.08)	.60
Aβ42	273	−0.03 (−0.61 to 0.56)	.93	188	−0.35 (−1.11 to 0.41)	.36	79	0.44 (−0.60 to 1.49)	.40
Aβ42/Aβ40 ratio	273	16.83 (−91.51 to 125.16)	.76	188	−1.11 (−146.86 to 144.63)	.99	79	71.88 (−160.52 to 304.28)	.54
GFAP	277	−0.01 (−0.05 to 0.03)	.54	188	−0.00 (−0.05 to 0.04)	.83	83	−0.00 (−0.08 to 0.08)	.95
NFL	277	−0.03 (−0.09 to 0.02)	.20	188	−0.03 (−0.10 to 0.05)	.49	83	0.02 (−0.09 to 0.13)	.70
T-tau	277	−0.22 (−0.49 to 0.05)	.11	188	−0.34 (−0.66 to −0.02)	.04	83	0.28 (−0.26 to 0.81)	.31
P-tau_231_	276	0.09 (−0.18 to 0.36)	.52	187	0.00 (−0.51 to 0.51)	>.99	83	0.16 (−0.15 to 0.47)	.31
Processing speed									
Aβ40	278	−0.02 (−0.06 to 0.01)	.19	189	−0.04 (−0.09 to 0.01)	.15	82	−0.02 (−0.09 to 0.04)	.46
Aβ42	276	−0.28 (−0.88 to 0.31)	.35	189	−0.46 (−1.27 to 0.36)	.27	80	−0.29 (−1.34 to 0.76)	.58
Aβ42/Aβ40 ratio	276	47.23 (−72.50 to 166.95)	.44	189	50.86 (−101.85 to 203.57)	.51	80	96.17 (−206.17 to 398.52)	.53
GFAP	280	−0.02 (−0.06 to 0.03)	.47	189	−0.01 (−0.06 to 0.04)	.69	84	−0.05 (−0.14 to 0.04)	.25
NFL	280	−0.05 (−0.08 to −0.01)	.02	189	−0.05 (−0.09 to −0.01)	.03	84	−0.03 (−0.16 to 0.09)	.58
T-tau	280	0.10 (−0.21 to 0.40)	.53	189	0.06 (−0.29 to 0.41)	.74	84	0.22 (−0.47 to 0.92)	.52
P-tau_231_	279	−0.03 (−0.23 to 0.16)	.75	188	−0.05 (−0.59 to 0.49)	.85	84	−0.04 (−0.27 to 0.19)	.73
Attention									
Aβ40	261	−0.02 (−0.05 to 0.02)	.34	175	−0.01 (−0.05 to 0.04)	.80	79	−0.01 (−0.07 to 0.04)	.60
Aβ42	259	−0.32 (−0.84 to 0.19)	.22	175	−0.01 (−0.73 to 0.70)	.97	77	−0.50 (−1.34 to 0.34)	.24
Aβ42/Aβ40 ratio	259	27.44 (−82.34 to 137.21)	.62	175	122.70 (−23.26 to 268.65)	.10	77	−196.17 (−444.78 to 52.43)	.12
GFAP	263	0.02 (−0.02 to 0.05)	.39	175	0.02 (−0.03 to 0.06)	.46	81	0.00 (−0.07 to 0.08)	.96
NFL	263	−0.04 (−0.08 to 0.01)	.09	175	−0.02 (−8.68 to 0.05)	.65	81	−0.04 (−0.14 to 0.06)	.45
T-tau	263	−0.00 (−0.26 to 0.26)	.98	175	−0.04 (−0.34 to 0.26)	.80	81	−0.00 (−0.57 to 0.56)	.99
P-tau_231_	262	−0.05 (−0.21 to 0.12)	.56	174	0.27 (−0.21 to 0.74)	.27	81	−0.09 (−0.28 to 0.10)	.35
Learning									
Aβ40	278	−0.06 (−0.09 to −0.02)	.002	190	−0.07 (−0.12 to −0.02)	.01	81	−0.06 (−0.12 to 0.01)	.08
Aβ42	276	−0.76 (−1.36 to −0.16)	.01	190	−0.80 (−1.64 to 0.03)	.06	79	−0.93 (−1.93 to 0.08)	.07
Aβ42/Aβ40	276	58.83 (−63.79 to 181.45)	.35	190	65.17 (−93.25 to 223.59)	.42	79	121.35 (−172.57 to 415.26)	.41
GFAP	280	−0.01 (−0.05 to 0.03)	.53	190	−0.01 (−0.06 to 0.04)	.65	83	−0.05 (−0.14 to 0.04)	.25
NFL	280	−0.01 (−0.05 to 0.03)	.57	190	−0.01 (−0.06 to 0.03)	.60	83	0.01 (−0.12 to 0.14)	.92
T-tau	280	−0.02 (−0.33 to 0.29)	.91	190	−0.04 (−0.41 to 0.33)	.84	83	−0.04 (−0.72 to 0.64)	.90
P-tau_231_	279	−0.11 (−0.31 to 0.08)	.25	189	−0.19 (−0.74 to 0.37)	.51	83	−0.15 (−0.38 to 0.08)	.19
Memory									
Aβ40	278	−0.05 (−0.09 to −0.01)	.006	190	−0.06 (−0.11 to −0.01)	.02	82	−0.07 (−0.13 to −0.01)	.03
Aβ42	276	−0.60 (−1.21 to 0.01)	.05	190	−0.57 (−1.42 to 0.27)	.18	79	−1.11 (−2.09 to −0.13)	.03
Aβ42/Aβ40 ratio	276	65.71 (−58.01 to 189.43)	.30	190	93.52 (−65.58 to 252.62)	.25	79	103.83 (−186.71 to 394.36)	.48
GFAP	280	−0.02 (−0.07 to 0.02)	.26	190	−0.03 (−0.08 to 0.02)	.25	83	−0.01 (−0.10 to 0.07)	.77
NFL	280	−0.01 (−0.05 to 0.03)	.74	190	−0.01 (−0.05 to 0.04)	.77	83	0.01 (−0.11 to 0.12)	.86
T-tau	280	−0.14 (−0.46 to 0.17)	.37	190	−0.17 (−0.54 to 0.20)	.36	83	−0.24 (−0.90 to 0.42)	.47
P-tau_231_	279	−0.17 (−0.36 to 0.03)	.10	189	−0.23 (−0.80 to 0.33)	.41	83	−0.21 (−0.43 to 0.01)	.06
Verbal fluency									
Aβ40	279	−0.02 (−0.05 to 0.02)	.29	190	−0.04 (−0.09 to 0.01)	.10	82	−0.01 (−0.06 to 0.04)	.84
Aβ42	277	−0.22 (−0.76 to 0.32)	.43	190	−0.47 (−1.24 to 0.30)	.23	80	−0.14 (−0.93 to 0.65)	.72
Aβ42/Aβ40 ratio	277	74.57 (−33.91 to 183.05)	.18	190	103.40 (−40.57 to 247.46)	.16	80	69.04 (−157.93 to 296.01)	.55
GFAP	281	−0.01 (−0.05 to 0.03)	.67	190	−0.00 (−0.05 to 0.05)	.98	84	−0.05 (−0.12 to 0.02)	.17
NFL	281	−0.04 (−0.07 to 0.00)	.04	190	−0.03 (−0.08 to 0.01)	.10	84	−0.03 (−0.13 to 0.06)	.47
T-tau	281	0.06 (−0.22 to 0.34)	.68	190	−0.03 (−0.36 to 0.31)	.88	84	0.19 (−0.34 to 0.73)	.47
P-tau_231_	280	−0.02 (−0.20 to 0.15)	.79	189	0.08 (−0.43 to 0.59)	.76	84	−0.03 (−0.21 to 0.15)	.72
Motor									
Aβ40	277	−0.04 (−0.08 to −0.00)	.04	188	−0.03 (−0.08 to 0.02)	.27	82	−0.07 (−0.14 to 0.01)	.09
Aβ42	275	−0.80 (−1.44 to −0.15)	.02	188	−0.80 (−1.67 to 0.07)	.07	80	−1.01 (−2.21 to 0.19)	.10
Aβ42/Aβ40 ratio	275	−72.56 (−203.84 to 58.71)	.28	188	−74.50 (−234.70 to 87.66)	.37	80	−128.78 (−482.39 to 224.82)	.47
GFAP	279	−0.03 (−0.08 to 0.01)	.15	188	−0.02 (−0.07 to 0.03)	.46	84	−0.09 (−0.19 to 0.02)	.11
NFL	279	−0.05 (−0.09 to −0.01)	.02	188	−0.02 (−0.06 to 0.03)	.44	84	−0.21 (−0.35 to −0.07)	.003
T-tau	279	−0.03 (−0.36 to 0.31)	.87	188	−0.02 (−0.40 to 0.36)	.92	84	0.21 (−0.61 to 1.02)	.62
P-tau_231_	278	−0.27 (−0.48 to −0.06)	.01	187	−0.55 (−1.11 to 0.02)	.06	84	−0.20 (−0.47 to 0.07)	.14
Global									
Aβ40	255	−0.02 (−0.05 to 0.00)	.09	173	−0.03 (−0.07 to −0.00)	.04	76	−0.00 (−0.05 to 0.04)	.85
Aβ42	253	−0.30 (−0.70 to 0.11)	.16	173	−0.51 (−1.03 to 0.02)	.06	74	−0.12 (−0.89 to 0.65)	.76
Aβ42/Aβ40 ratio	253	29.99 (−48.33 to 108.30)	.45	173	38.50 (−69.47 to 146.50)	.48	74	−11.58 (−185.17 to 162.01)	.89
GFAP	257	−0.01 (−0.03 to 0.02)	.60	173	−0.01 (−0.04 to 0.02)	.60	78	0.00 (−0.05 to 0.06)	.90
NFL	257	−0.03 (−0.06 to 0.01)	.13	173	−0.03 (−0.08 to 0.02)	.29	78	0.00 (−0.08 to 0.08)	.99
T-tau	257	−0.05 (−0.23 to 0.14)	.63	173	−0.10 (−0.32 to 0.12)	.36	78	0.13 (−0.25 to 0.51)	.50
P-tau_231_	256	0.06 (−0.13 to 0.25)	.54	172	−0.02 (−0.37 to 0.32)	.89	78	0.07 (−0.15 to 0.29)	.54

Abbreviations: Aβ, amyloid β; AD, Alzheimer disease; GFAP, glial fibrillary protein; NFL, neurofilament light chain; P-tau, phosphorylated tau; T-tau, total tau; WLWH, women living with HIV; WLWOH, women living without HIV.

^a^ All models are adjusted for HIV status, income, body mass index, Center for Epidemiological Studies-Depression scale score, eGFR estimated glomerular filtration rate, history of hypertension, diabetes, tobacco use, marijuana use, alcohol use, and other drug use (crack, cocaine, or heroin). β represents an adjusted, unstandardized coefficient. Neuropsychological performance assessments were reverse scored and a higher β indicates better neuropsychological performance.

^b^ WLWH models were additionally adjusted for baseline CD4 T cell count, HIV viral load, and history of AIDS.

## Discussion

In this cohort study, we found that among WLWH and WLWOH with similar sociodemographic characteristics, 1-year increases in plasma Aβ40, Aβ42, p-tau_231_, and NFL were associated with worse motor performance. The association of 1-year increase in plasma Aβ40 with memory performance was consistent among both WLWH and WLWOH, and the magnitude of the regression coefficient was largest for NP associations with Aβ42 followed by t-tau and p-tau_231_. In addition, WLWH performed more poorly on learning, memory, verbal fluency and global NP compared with WLWOH, as previously reported in the WIHS.^14,29,30,34^

The plasma biomarkers measured reflect the ATN criteria for AD^10^ and differentiate AD from other types of dementia.^8,35^ However, traditionally, cerebrospinal fluid (CSF) measures of these biomarkers have been used to make this differentiation. Associations of CSF with some plasma biomarkers are not robust and none reach a perfect association.^9^ In addition, other hypotheses related to AD risk and pathology have been proposed including those related to vascular pathologies, inflammation, neuroimmune dysregulation, synaptic dysfunction, blood-brain barrier alternations, and communicable disease reservoirs.^11,12^ The construct of multiple-etiology dementias needs to be addressed, and understanding these hypotheses in underrepresented communities with health disparities is underscored.^36^

The majority of our associations spanning 5 cognitive domains were observed with 1-year change in 2 plasma Aβ peptides (Aβ40 and Aβ42) and NFL in the entire sample adjusted for HIV status and other covariates. In addition, worse motor performance was consistent with 1-year increase in these 3 biomarkers, as well as p-tau_231_. Aβ peptides are cleavage products of amyloid precursor protein, a transmembrane protein present in almost every cell of the human body. Aβ deposits (amyloid plaques) in the brain are a neuropathological hallmark of both clinical and neuropathological AD.^10^ Lower levels of Aβ42 and Aβ40 in CSF and plasma and decreases in those levels are associated with AD,^37^ cognitive decline,^38^ and psychiatric and behavioral symptoms in aging individuals without HIV.^39^ Some studies have reported no association with either plasma Aβ42 or Aβ40 levels in PLWH, specifically men who have sex with men and transgender women.^40^

We observed no association of NP with the Aβ42-to-Aβ40 ratio, which has been associated with an increased incidence of AD in HIV-uninfected aging individuals.^9^ However, no association of plasma Aβ42-to-Aβ40 ratio in people aged older than 40 years with AD and without HIV compared with similar-aged PLWH who were cognitively normal or who had AD was also reported.^41^

Tau biomarkers in CSF, notably t-tau and p-tau, are prognostic and diagnostic for AD, which is characterized by neurofibrillary tangles.^42^ Recently, tau positron emission tomography has evolved for clinical diagnosis of AD and differentiates from other neurodegenerative tauopathies.^43^ P-tau_231_ may be the preferred CSF discriminatory tau protein for an AD diagnosis.^43,44^ We observed 1 association of p-tau_231 _with motor performance. To our knowledge, there are no published reports on this blood-based protein in PLWH.

Neurofilaments are neuronal cytoskeletal proteins released into the CSF or blood in response to neurological injuries and neurodegeneration and have been associated with dementia, stroke, and traumatic brain injury. HIV crosses the blood-brain barrier, activates microglia, and leads to neuroinflammation and neurodegeneration, resulting in the release of NFL.^45^ Published studies^46^ in cross-sectional samples of PLWH have reported higher NFL levels in association with lower NP, and no association between baseline levels of NFL and subsequent cognitive decline over 24 months or more in PLWH, including men who have sex with men and transgender women.^40,47^ Some reports indicate that impaired kidney function, more commonly observed among PLWH, may be associated with changes in blood NFL levels.^33^ However, since the molecular weight of NFL is more than 60 kDa and NFL is negatively charged, its biokinetics may be unaffected in people with impaired kidney function as measured via eGFR.^48^

We observed no associations of NP with GFAP, even though astrocytes make up approximately30% of the cells in the central nervous system and are constituents of the blood-brain barrier.^49^ GFAP is an astrocyte protein measurable in CSF and plasma and is a biomarker of both central neurological disorders and injuries.^9^

Our analyses have many strengths. First, the WIHS cohort was active with a consistent research protocol for over 25 years. Second, the WIHS comprises WLWH and WLWOH who are underrepresented in research. The cohort consisted of women of many races and ethnicities and predominately included women who have lower educational attainment and lower annual income. Third, we used state-of-the-art Simoa technology to analyze our biomarker samples. Single molecule protein detection is based on the isolation of individual immunocomplexes on paramagnetic beads using standard enzyme-linked immunosorbent assay reagents and is 1000 times more sensitive than traditional enzyme-linked immunosorbent assays. However, differences in laboratory methods and evolution in sensitivity and specificity over time in this research field may contribute to differences in published results. Fourth, to our knowledge, there are no other publications presenting data based on a cohort of WLWH and comparison group of similar women assessing 6 blood-based AD biomarkers repeated over a 1-year period in association with NP assessments. Fifth, Aβ isoforms, such as Aβ42 and Aβ40, and t-tau and p-tau isoforms have traditionally been measured in the CSF. However, CSF collection is not practical in many underrepresented communities. Finally, due to extensive clinical data available in the WIHS, we were able to adjust for eGFR; however, this adjustment did not influence the results in all essential aspects.

### Limitations

As with any epidemiologic observational study, there are limitations to our analyses. First, although the study demonstrated several associations of NP with blood-based biomarkers, external validity may be compromised. WIHS participants in these analyses comprised a well-studied group of women who were followed for 6 to 7 years or more. Second, since WIHS was an intent-to-treat cohort, these women may have been healthier than WLWH who did not participate in a research study. In 2018 and 2019, at the time of these blood measures, more than 90% reported being adherent to ART.^13^ Although ARTs are associated with lower blood levels of NFL,^50^ the WLWH in our study had higher NFL levels compared with WLWOH. Third, some of the biomarkers measured are sensitive but not specific. For example, higher blood levels of NFL and GFAP have been observed in several neurological conditions and accompany peripheral and central neurological injury or events.^13^ Fourth, other blood-based biomarkers of inflammation (eg, chemokines, interleukins, and tissue inhibitor of metalloproteinases-1) and altered metabolism (eg, leptin, adiponectin, ghrelin, amylin, and gastric inhibitory peptide) have been associated with NP in WLWH in the WIHS at different times over the adult life course.^14,29,32^ We cannot consider their contribution here. However, other biomarker study results have been consistent with HIV pathophysiology and associated comorbidities, such as obesity, and are potential biomarkers that may be useful in the creation of a biomarker panel among WLWH with promising clinical applications. Fifth, our sample is composed primarily of Black women predominantly living in urban environments; thus, extrapolation to other racial and ethnic groups, men, and living environments is limited. Sixth, EDTA plasma is required for all AD and related neurodegenerative disorder biomarkers measured via Simoa except for NFL. In our cohort, we did not collect EDTA plasma until 2018. In combination with NP assessments being given once every 2 years, we can only evaluate a 1-year biomarker change in association with 1 NP assessment. Seventh, due to the exploratory nature of our analyses, we chose not to adjust for multiple comparisons. Therefore, some of our results may represent type 1 error. Eighth, data on other neurological symptoms, signs, and outcomes (eg, stroke) that may be associated with NP were not routinely collected in this cohort or were rare.

## Conclusion

This study found that increases in plasma Aβ42 and Aβ40 peptides, p-tau_231_, and NFL over time were associated with NP. These findings suggest that they are promising preclinical biomarkers of NP and brain health in underrepresented samples of WLWH and WLWOH.

## Notes

Supplement 1. **eFigure 1.** Association Between Baseline and Follow-Up Levels of Aβ40 in the Entire Sample **eFigure 2.** Association Between Baseline and Follow-Up Levels of Aβ42 in the Entire Sample **eFigure 3.** Association Between Baseline and Follow-Up Levels of GFAP in the Entire Sample **eFigure 4.** Association Between Baseline and Follow-Up Levels of NFL in the Entire Sample **eFigure 5.** Association Between Baseline and Follow-Up Levels of T-tau in the Entire Sample **eFigure 6.** Association Between Baseline and Follow-Up Levels of P-tau231 in the Entire Sample

Supplement 2.
Data Sharing Statement

## References

[1] Bosh KA, Johnson AS, Hernandez AL, . Vital signs: deaths among persons with diagnosed HIV Infection, United States, 2010-2018. MMWR Morb Mortal Wkly Rep. 2020;69(46):1717-1724. doi:10.15585/mmwr.mm6946a1 33211683 PMC7676640

[2] World Health Organization. HIV. 2023. Accessed March 22, 2023. https://www.who.int/data/gho/data/themes/hiv-aids

[3] Joseph SB, Gianella S, Burdo TH, . Biotypes of central nervous system complications in people with human immunodeficiency virus: virology, immunology, and neuropathology. J Infect Dis. 2023;227(suppl 1):S3-S15. doi:10.1093/infdis/jiac370 36930640 PMC10022721

[4] Bandera A, Taramasso L, Bozzi G, . HIV-associated neurocognitive impairment in the modern ART era: are we close to discovering reliable biomarkers in the setting of virological suppression? Front Aging Neurosci. 2019;11:187. doi:10.3389/fnagi.2019.00187 31427955 PMC6687760

[5] 2023 Alzheimer’s disease facts and figures. Alzheimers Dement. 2023;19(4):1598-1695. doi:10.1002/alz.13016 36918389

[6] World Health Organization. The top 10 causes of death. 2023. Accessed March 22, 2023. https://www.who.int/news-room/fact-sheets/detail/the-top-10-causes-of-death

[7] Winston A, Spudich S. Cognitive disorders in people living with HIV. Lancet HIV. 2020;7(7):e504-e513. doi:10.1016/S2352-3018(20)30107-7 32621876

[8] Jack CR Jr, Bennett DA, Blennow K, . NIA-AA research framework: toward a biological definition of Alzheimer’s disease. Alzheimers Dement. 2018;14(4):535-562. doi:10.1016/j.jalz.2018.02.018 29653606 PMC5958625

[9] Zetterberg H. Biofluid-based biomarkers for Alzheimer’s disease-related pathologies: an update and synthesis of the literature. Alzheimers Dement. 2022;18(9):1687-1693. doi:10.1002/alz.12618 35213777 PMC9514308

[10] Jack CR Jr, Bennett DA, Blennow K, . A/T/N: an unbiased descriptive classification scheme for Alzheimer disease biomarkers. Neurology. 2016;87(5):539-547. doi:10.1212/WNL.0000000000002923 27371494 PMC4970664

[11] Hampel H, Cummings J, Blennow K, Gao P, Jack CR Jr, Vergallo A. Developing the ATX(N) classification for use across the Alzheimer disease continuum. Nat Rev Neurol. 2021;17(9):580-589. doi:10.1038/s41582-021-00520-w 34239130

[12] Sachdev PS. Developing robust biomarkers for vascular cognitive disorders: adding ‘V’ to the AT(N) research framework. Curr Opin Psychiatry. 2020;33(2):148-155. doi:10.1097/YCO.0000000000000577 31895155

[13] Adimora AA, Ramirez C, Benning L, . Cohort profile: the women’s interagency HIV Study (WIHS). Int J Epidemiol. 2018;47(2):393-394i. doi:10.1093/ije/dyy021 29688497 PMC5913596

[14] Gustafson DR, Mielke MM, Keating SA, Holman S, Minkoff H, Crystal HA. Leptin, adiponectin and cognition in middle-aged HIV-infected and uninfected women: the Brooklyn women’s interagency HIV study. J Gerontol Geriatr Res. 2015;4(5):240. doi:10.4172/2167-7182.1000240 27536467 PMC4984413

[15] National Heart, Lung, and Blood Institute. Classification of overweight and obesity by BMI, waist circumference, and associated disease risks. US Department of Health and Human Services. Accessed February 4, 2023. https://www.nhlbi.nih.gov/health/educational/lose_wt/BMI/bmi_dis.htm

[16] Galaviz KI, Schneider MF, Tien PC, . Predicting diabetes risk among HIV-positive and HIV-negative women. AIDS. 2018;32(18):2767-2775. doi:10.1097/QAD.0000000000002017 30289812 PMC6673643

[17] Levey AS, Stevens LA, Schmid CH, ; CKD-EPI (Chronic Kidney Disease Epidemiology Collaboration). A new equation to estimate glomerular filtration rate. Ann Intern Med. 2009;150(9):604-612. doi:10.7326/0003-4819-150-9-200905050-00006 19414839 PMC2763564

[18] Radloff LS. The CES-D scale: a self report depression scale for research in the general population. Appl Psychol Meas. 1977;1(3):385-401. doi:10.1177/014662167700100306

[19] Wechsler D. WAIS-III Administration and Scoring Manual: Wechsler Adult Intelligence Scale. The Psychological Corporation; 1997.

[20] Wechsler D. WAIS-IV Technical and Interpretive Manual. Pearson, Inc; 2008.

[21] Reitan RM, Wolfson D. The Halstead–Reitan Neuropsycholgical Test Battery: Therapy and Clinical Interpretation. Neuropsychological Press; 2008.

[22] Comalli PE Jr, Wapner S, Werner H. Interference effects of Stroop color-word test in childhood, adulthood, and aging. J Genet Psychol. 1962;100:47-53. doi:10.1080/00221325.1962.10533572 13880724

[23] Benedict RHB, Schretlen D, Groninger L, Brandt J. Hopkins Verbal Learning Test--Revised (HVLT-R). APA PsycTests; 1991.

[24] Smith A. The symbol-digit modalities test: a neuropsychologic test of learning and other cerebral disorders. In: Helmuth J, ed. Learning Disorders. Special Child Publications; 1968:83-91.

[25] Smith A. Symbol Digits Modalities Test. Western Psychological Services; 1982.

[26] Benton AL, Hamsher K, Sivan AB. Multilingual Aphasia Examination. 3rd ed. AJA Associates; 1994.

[27] Brickman AM, Paul RH, Cohen RA, . Category and letter verbal fluency across the adult lifespan: relationship to EEG theta power. Arch Clin Neuropsychol. 2005;20(5):561-573. doi:10.1016/j.acn.2004.12.006 15939182 PMC2758771

[28] Ruff RM, Parker SB. Gender- and age-specific changes in motor speed and eye-hand coordination in adults: normative values for the Finger Tapping and Grooved Pegboard Tests. Percept Mot Skills. 1993;76(3 Pt 2):1219-1230. doi:10.2466/pms.1993.76.3c.1219 8337069

[29] Macaluso F, Weber KM, Rubin LH, . Body mass index and leptin are related to cognitive performance over 10 years in women with and without HIV infection. J Clin Endocrinol Metab. 2022;107(3):e1126-e1135. doi:10.1210/clinem/dgab759 34677589 PMC8851924

[30] Rubin LH, Radtke KK, Eum S, . Cognitive burden of common non-antiretroviral medications in HIV-infected women. J Acquir Immune Defic Syndr. 2018;79(1):83-91. doi:10.1097/QAI.0000000000001755 29781879 PMC6092212

[31] Ashton NJ, Pascoal TA, Karikari TK, . Plasma p-tau231: a new biomarker for incipient Alzheimer’s disease pathology. Acta Neuropathol. 2021;141(5):709-724. doi:10.1007/s00401-021-02275-6 33585983 PMC8043944

[32] McFarlane SI, Mielke MM, Uglialoro A, . Ghrelin, amylin, gastric inhibitory peptide and cognition in middle-aged HIV-infected and uninfected women: the women’s interagency HIV study. J Neurol Neurophysiol. 2017;8(1):413. doi:10.4172/2155-9562.1000413 28690913 PMC5497768

[33] Syrjanen JA, Campbell MR, Algeciras-Schimnich A, . Associations of amyloid and neurodegeneration plasma biomarkers with comorbidities. Alzheimers Dement. 2022;18(6):1128-1140. doi:10.1002/alz.12466 34569696 PMC8957642

[34] Rubin LH, Maki PM, Springer G, ; Women’s Interagency HIV Study. Cognitive trajectories over 4 years among HIV-infected women with optimal viral suppression. Neurology. 2017;89(15):1594-1603. doi:10.1212/WNL.0000000000004491 28904086 PMC5634661

[35] Benussi A, Cantoni V, Rivolta J, . Classification accuracy of blood-based and neurophysiological markers in the differential diagnosis of Alzheimer’s disease and frontotemporal lobar degeneration. Alzheimers Res Ther. 2022;14(1):155. doi:10.1186/s13195-022-01094-5 36229847 PMC9558959

[36] Rost NS. ADRD summit 2022 Report to the national advisory neurological disorders and stroke council. September 2022. National Institute of Neurological Disoders and Stroke. Accessed October 20, 2023. https://www.ninds.nih.gov/news-events/events/adrd-summit-2022

[37] Chatterjee P, Pedrini S, Doecke JD, . Plasma Aβ42/40 ratio, p-tau181, GFAP, and NfL across the Alzheimer’s disease continuum: a cross-sectional and longitudinal study in the AIBL cohort. Alzheimers Dement. 2022;19(4):1117-1134. doi:10.1002/alz.12724 36574591

[38] Gustafson DR, Skoog I, Rosengren L, Zetterberg H, Blennow K. Cerebrospinal fluid beta-amyloid 1-42 concentration may predict cognitive decline in older women. J Neurol Neurosurg Psychiatry. 2007;78(5):461-464. doi:10.1136/jnnp.2006.100529 17098843 PMC2117838

[39] Gudmundsson P, Skoog I, Waern M, . Is there a CSF biomarker profile related to depression in elderly women? Psychiatry Res. 2010;176(2-3):174-178. doi:10.1016/j.psychres.2008.11.012 20132991

[40] Longino AA, Paul R, Wang Y, . HIV disease dynamics and markers of inflammation and CNS injury during primary HIV infection and their relationship to cognitive performance. J Acquir Immune Defic Syndr. 2022;89(2):183-190. doi:10.1097/QAI.0000000000002832 34629415 PMC8752485

[41] Cooley SA, Nelson B, Boerwinkle A, . Plasma Aβ42/Aβ40 ratios are not reduced in older people living with HIV. Clin Infect Dis. 2023;76(10):1776-1783. doi:10.1093/cid/ciad001 36610788 PMC10209437

[42] Braak H, Braak E. Neuropathological stageing of Alzheimer-related changes. Acta Neuropathol. 1991;82(4):239-259. doi:10.1007/BF00308809 1759558

[43] Wang YT, Edison P. Tau imaging in neurodegenerative diseases using positron emission tomography. Curr Neurol Neurosci Rep. 2019;19(7):45. doi:10.1007/s11910-019-0962-7 31172290 PMC6554240

[44] Spiegel J, Pirraglia E, Osorio RS, . Greater specificity for cerebrospinal fluid P-tau231 over P-tau181 in the differentiation of healthy controls from Alzheimer’s disease. J Alzheimers Dis. 2016;49(1):93-100. doi:10.3233/JAD-150167 26444757 PMC4694576

[45] Borrajo A, Spuch C, Penedo MA, Olivares JM, Agís-Balboa RC. Important role of microglia in HIV-1 associated neurocognitive disorders and the molecular pathways implicated in its pathogenesis. Ann Med. 2021;53(1):43-69. doi:10.1080/07853890.2020.1814962 32841065 PMC7877929

[46] Anderson AM, Easley KA, Kasher N, . Neurofilament light chain in blood is negatively associated with neuropsychological performance in HIV-infected adults and declines with initiation of antiretroviral therapy. J Neurovirol. 2018;24(6):695-701. doi:10.1007/s13365-018-0664-y 30105502 PMC6279552

[47] Anderson AM, Jang JH, Easley KA, . Cognitive and neuronal link with inflammation: a longitudinal study in people with and without HIV infection. J Acquir Immune Defic Syndr. 2020;85(5):617-625. doi:10.1097/QAI.0000000000002484 32932412 PMC8725606

[48] Alagaratnam J, De Francesco D, Zetterberg H, . Correlation between cerebrospinal fluid and plasma neurofilament light protein in treated HIV infection: results from the COBRA study. J Neurovirol. 2022;28(1):54-63. doi:10.1007/s13365-021-01026-3 34874540 PMC9076742

[49] Sweeney MD, Sagare AP, Zlokovic BV. Blood-brain barrier breakdown in Alzheimer disease and other neurodegenerative disorders. Nat Rev Neurol. 2018;14(3):133-150. doi:10.1038/nrneurol.2017.188 29377008 PMC5829048

[50] Hagberg L, Edén A, Zetterberg H, Price RW, Gisslén M. Blood biomarkers for HIV infection with focus on neurologic complications-a review. Acta Neurol Scand. 2022;146(1):56-60. doi:10.1111/ane.13629 35470863 PMC9324809

