# Error in Byline

**DOI:** 10.1001/jamanetworkopen.2023.53563

**Published:** 2024-01-09

In the Original Investigation titled “Plasma Biomarkers of Alzheimer Disease in Women With and Without HIV,”^1^ published Novmber 29, 2023, there was an error for an author’s name in the byline, which should have appeared as Lauren F. Collins, MD. This article has been corrected.^1^
