# Supplementary material for: Plasma Biomarkers of Alzheimer Disease in Women With and Without HIV
Source: JAMA Netw Open. 2023 Nov 29;6(11):e2344194. doi: 10.1001/jamanetworkopen.2023.44194 (PMC10687654; doi:10.1001/jamanetworkopen.2023.44194)
Supplement: Supplement 1. — eFigure 1. Association Between Baseline and Follow-Up Levels of Aβ40 in the Entire Sample eFigure 2. Association Between Baseline and Follow-Up Levels of Aβ42 in the Entire Sample eFigure 3. Association Between Baseline and Follow-Up Levels of GFAP in the Entire Sample eFigure 4. Association Between Baseline and Follow-Up Levels of NFL in the Entire Sample eFigure 5. Association Between Baseline and Follow-Up Levels of T-tau in the Entire Sample eFigure 6. Association Between Baseline and Follow-Up Levels of P-tau231 in the Entire Sample [file jamanetwopen-e2344194-s001.pdf]

Supplemental Online Content

Li X, Yucel R, Clervius H, et al. Plasma Biomarkers of Alzheimer Disease in Women With and Without HIV. JAMA Netw Open. 2023;6(11):e2344194. doi:10.1001/jamanetworkopen.2023.44194

eFigure 1. Association Between Baseline and Follow-Up Levels of Aβ40 in the Entire Sample

eFigure 2. Association Between Baseline and Follow-Up Levels of Aβ42 in the Entire Sample

eFigure 3. Association Between Baseline and Follow-Up Levels of GFAP in the Entire Sample

eFigure 4. Association Between Baseline and Follow-Up Levels of NFL in the Entire Sample

eFigure 5. Association Between Baseline and Follow-Up Levels of T-tau in the Entire Sample

eFigure 6. Association Between Baseline and Follow-Up Levels of P-tau231 in the Entire Sample

This supplemental material has been provided by the authors to give readers additional information about their work.

© 2023 Li X et al. *JAMA Network Open*

eFigure 1. Association Between Baseline and Follow-Up Levels of Aβ40 in the Entire Sample

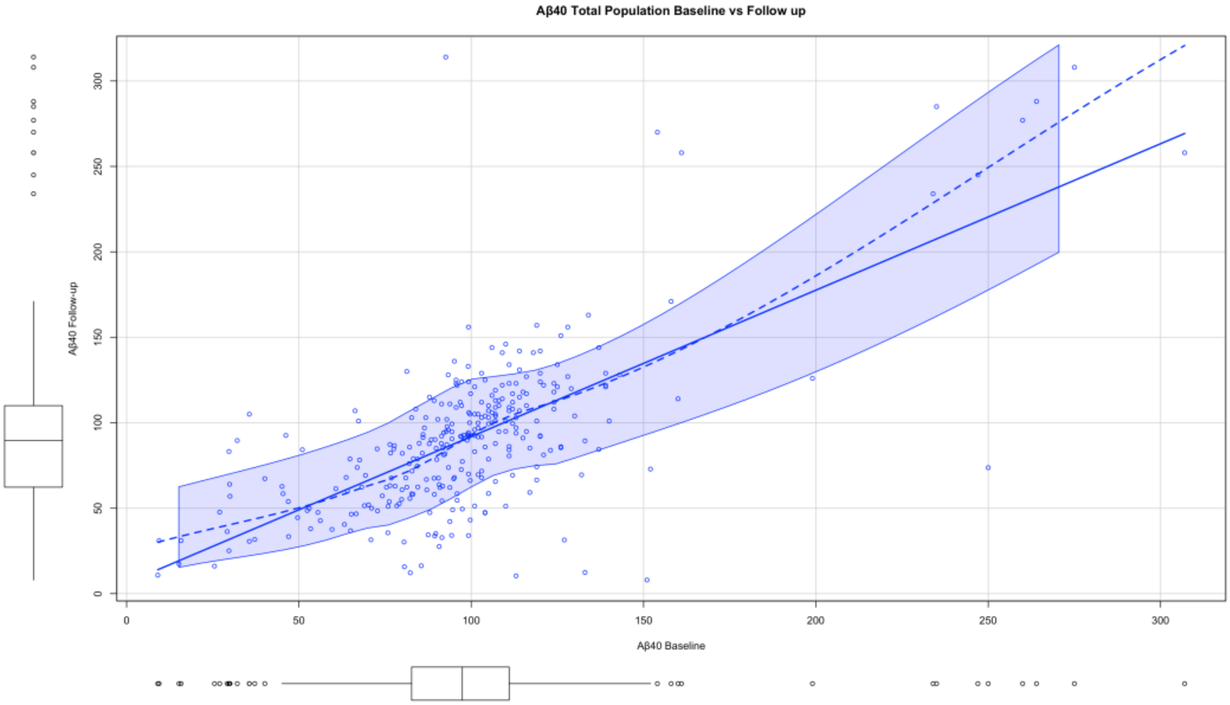

Baseline Aβ40 by follow-up Aβ40 levels (pg/mL)

© 2023 Li X et al. *JAMA Network Open*

eFigure 2. Association Between Baseline and Follow-Up Levels of Aβ42 in the Entire Sample

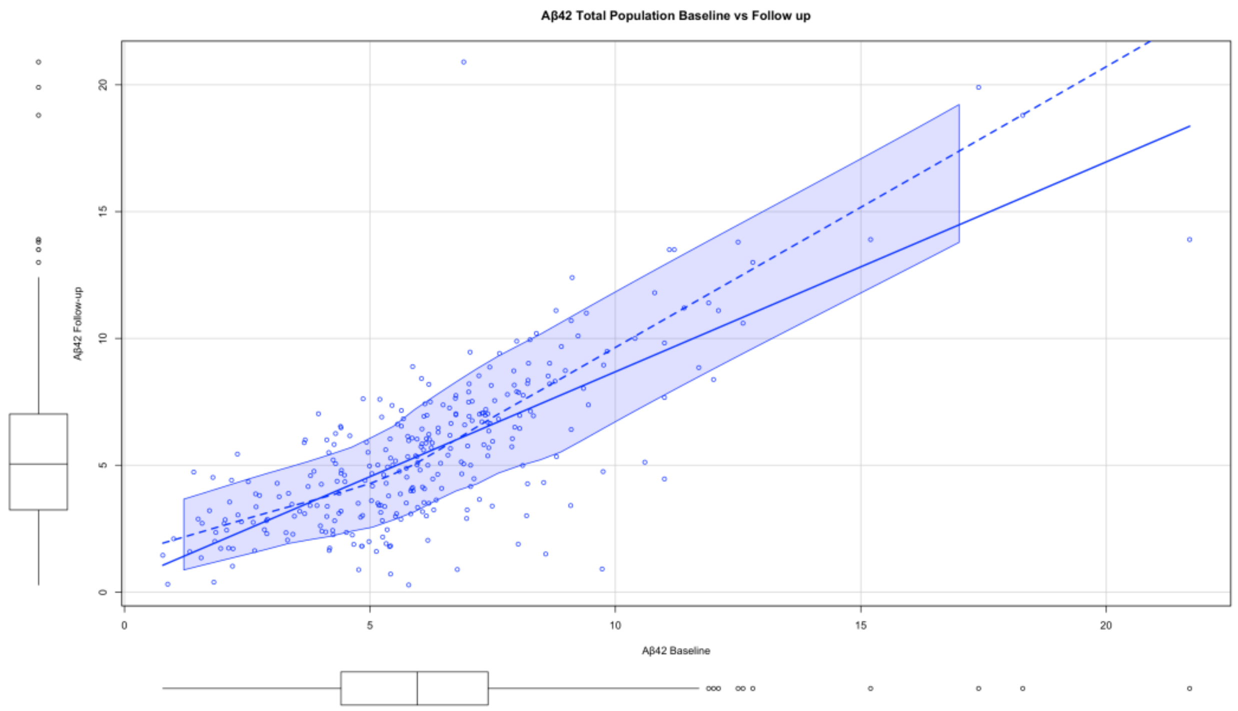

Baseline Aβ42 by Aβ42 level at follow-up (pg/mL)

© 2023 Li X et al. *JAMA Network Open*

eFigure 3. Association Between Baseline and Follow-Up Levels of GFAP in the Entire Sample

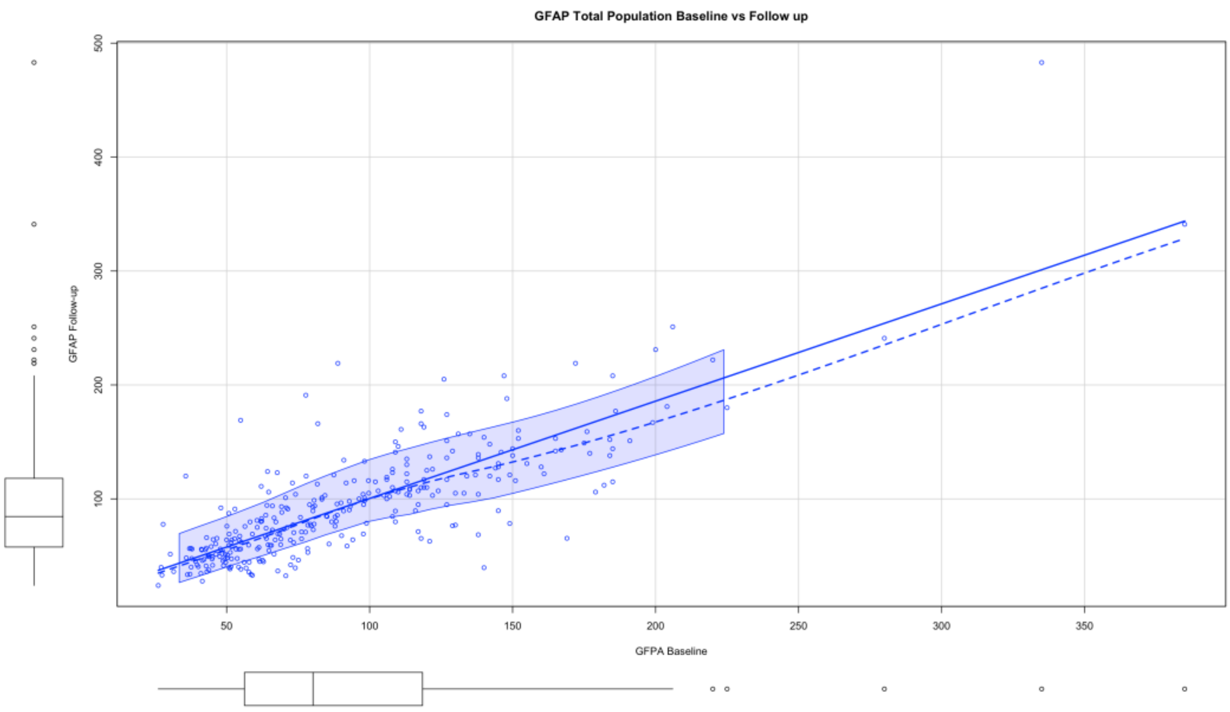

Baseline GFP by GFAP level at follow-up (pg/mL)

© 2023 Li X et al. *JAMA Network Open*

eFigure 4. Association Between Baseline and Follow-Up Levels of NFL in the Entire Sample

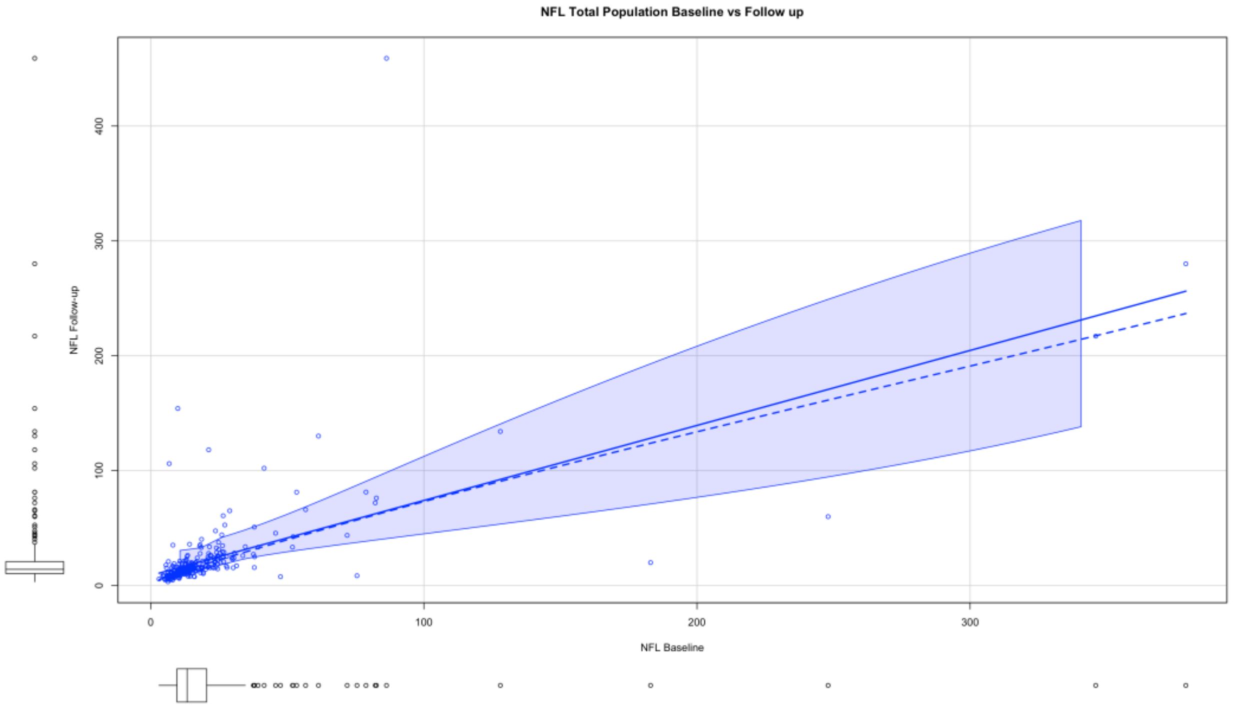

Baseline NFL by NFL at follow-up (pg/mL)

© 2023 Li X et al. *JAMA Network Open*

eFigure 5. Association Between Baseline and Follow-Up Levels of T-tau in the Entire Sample

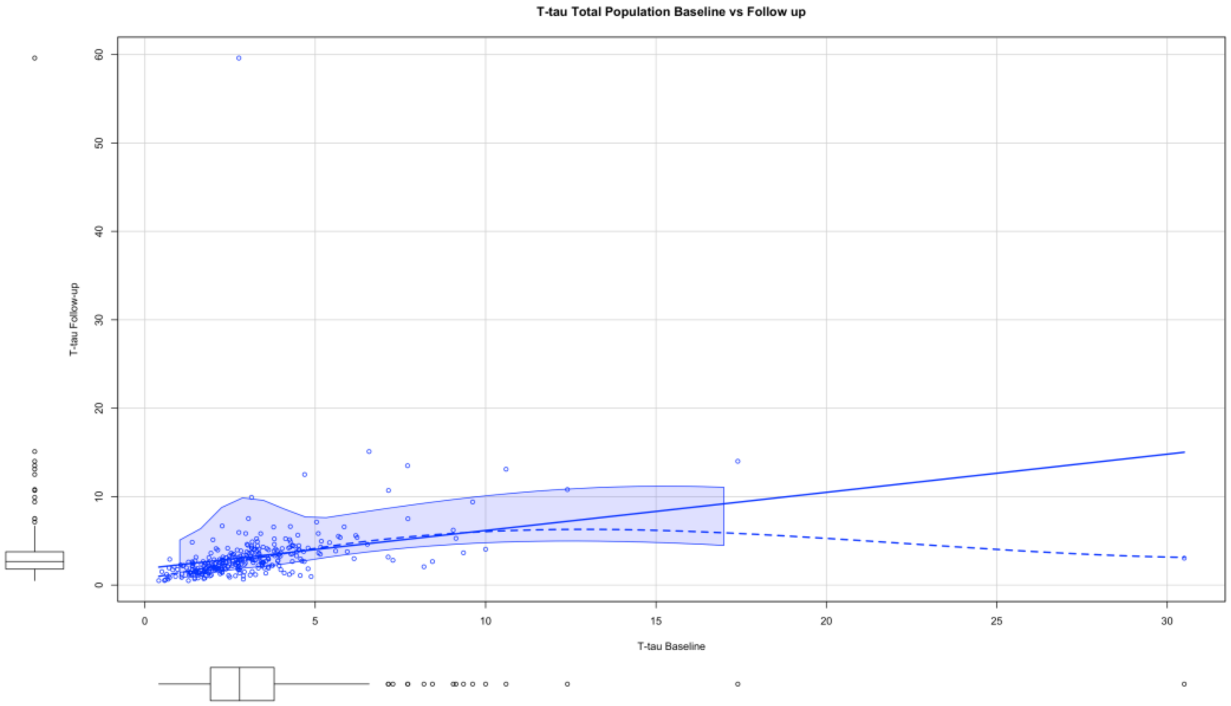

Baseline T-tau by T-tau at follow-up (pg/mL)

© 2023 Li X et al. *JAMA Network Open*

eFigure 6. Association Between Baseline and Follow-Up Levels of P-tau231 in the Entire Sample

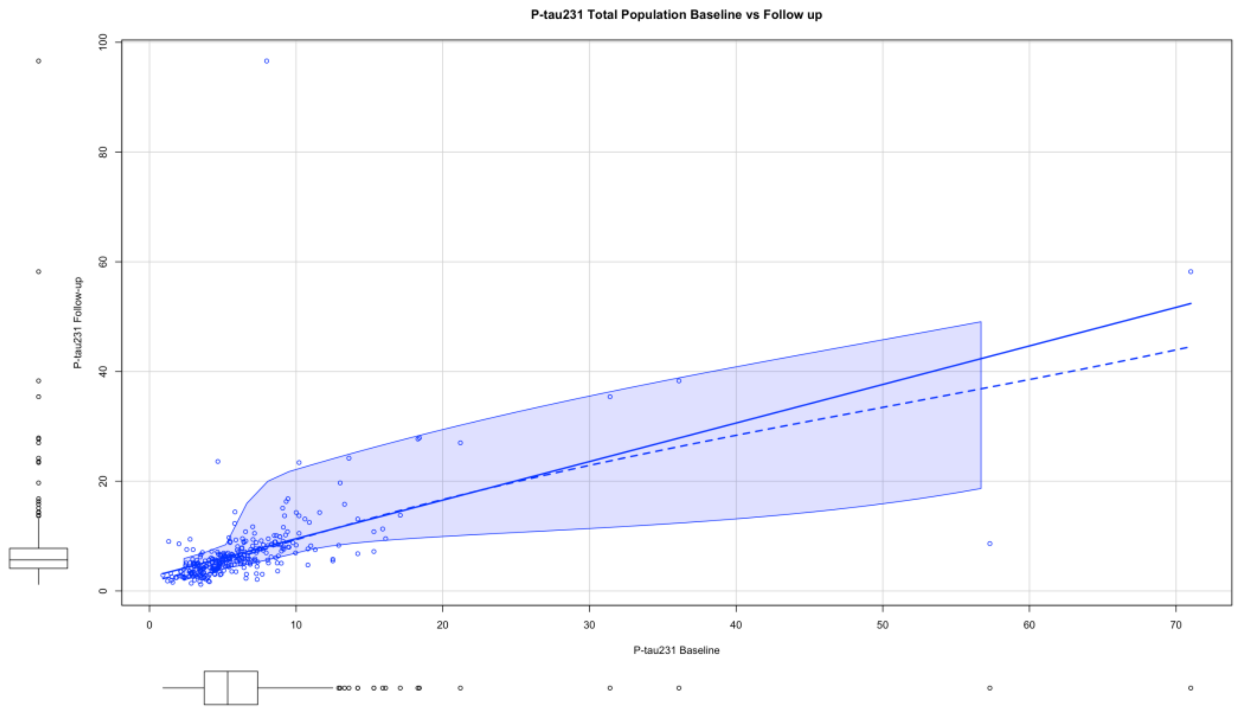

Baseline P-tau231 by P-tau231 at follow-up (pg/mL)

© 2023 Li X et al. *JAMA Network Open*
