# Supplementary material for: Plasma Biomarkers of Alzheimer Disease in Women With and Without HIV
Source: JAMA Netw Open. 2023 Nov 29;6(11):e2344194. doi: 10.1001/jamanetworkopen.2023.44194 (PMC10687654; doi:10.1001/jamanetworkopen.2023.44194)
Supplement: Supplement 2. — Data Sharing Statement [file jamanetwopen-e2344194-s002.pdf]

Data Sharing Statement

Li. Plasma Biomarkers of Alzheimer Disease in Women With and Without HIV. *JAMA Netw Open*. Published November 29, 2023. doi:10.1001/jamanetworkopen.2023.44194

Data
Data available: Yes
Data types: Deidentified participant data
How to access data: Data will be available upon request from the Principal Investigator upon submission of a study Concept Sheet.
When available: With publication

Supporting Documents
Document types: Statistical/analytic code
How to access documents: Deborah.Gustafson@downstate.edu after following the guidelines found here: https://statepi.jhsph.edu/mwccs/work-with-us/
When available: With publication

Additional Information
Who can access the data: Researchers whose proposed use of the data has been approved by MWCCS investigators.
Types of analyses: Specified purpose
Mechanisms of data availability: After approval of a proposal, i.e., an MWCCS Concept Sheet. Instructions can be found here: https://statepi.jhsph.edu/mwccs/work-with-us/
Any additional restrictions: NA
